# Personal values influencing career path in academic medicine: Perspectives of selected Canadian trainees

**DOI:** 10.12688/f1000research.9026.2

**Published:** 2018-04-25

**Authors:** Marissa Tsoi, Braden D. Teitge, Christopher R. Madan, Louis H. Francescutti

**Affiliations:** 1 Department of Family & Community Medicine, University of Toronto, Toronto, Canada; 2 Cumming School of Medicine, University of Calgary, Calgary, Canada; 3 Department of Psychology, Boston College, Chestnut Hill, USA; 4 School of Psychology, University of Nottingham, Nottingham, UK; 5 School of Public Health, University of Alberta, Edmonton, Canada

**Keywords:** medical education, academic medicine, personal values, MD/PhD, Canadian medical education, undergraduate medical education, postgraduate medical education, continuous medical education

## Abstract

To pursue research, education, and health policy in one’s career, broadly defined as academic medicine, is one of the most important decisions of a trainee doctor’s career. Despite this, there is scant literature on which factors influence trainees’ choices towards clinical work or academic research. As the MD/PhD is a relatively young training path compared to the traditional PhD (Doctor of Philosophy) and MD (Doctor of Medicine) programs, it prompts the question: at the crossroads of a career, what sways the individual to select an MD, PhD, or MD/PhD program? This is a valuable question to be answered for trainees who are considering multiple career paths, for educators who want to guide undifferentiated students, and for policy makers who develop and coordinate research programs. “Intellectual stimulation” is the most consistently identified personal value which draws trainees to academic medicine. Mentorship is linked strongly to success in the field. Conversely, long training periods, a lack of autonomy, and financial considerations are deterrents from a career in academic medicine. Insight into the decision-making process is provided by recent Canadian trainees in these respective fields, in a series of short interviews.

## What is academic medicine, where does it start, and who chooses it?

Academic medicine is broadly defined as “the discovery and development of basic principles, effective policies, and best practices that advance research and education in the health sciences, ultimately to improve the health and well-being of individuals and populations” ^1^. The interest in academic medicine comes from the fundamental tenet of modern medicine based on discovery, research and innovation. Physicians that are active in research and innovation help to keep medical knowledge and clinical care on the cutting edge, constantly improving and ensuring that we deliver the best care for our patients. Healthcare policy makers too must be familiar with health policy research to guide their decision-making and academic activity.

Encouraging the next generation of physicians to become active in research and health policy can only help the advancement of clinical care, from the bench to the bedside. Trainees may undertake academic medicine at several points in their career. Many obtain MSc or PhD degrees prior to enrolling in medical school; others enroll in a combined MD/PhD program or complete a PhD degree during residency training; others yet decide later, as independent physicians, to add an academic component to their practice. Many residencies incorporate a strong academic aspect, especially for those completing fellowships in a sub-specialty.

With respect to the germination of this interest, it is recognized that research interest often begins in residency and medical school, with early exposure to research in medical school fostering interest in it as a career ^2^. However, many factors determining career path are at play even earlier on, before the start of a graduate program or acceptance into a medical school. By virtue of entrance criteria, a decision whether to enter an MD or graduate program likely occurs around the time of undergraduate degree completion. Furthermore, it is interesting to examine the ongoing decision to remain in a chosen career pathway, for research has shown that trainees’ interest in academic medicine wanes as they progress ^2^.

Due to the various routes to a career in academic medicine, it is difficult to define distinct decision points in time when a trainee chooses to pursue academic medicine. Many extrinsic factors which influence career choice may vary over the course of this journey; this makes trainees' personal values relevant, if regarded as a consistent factor. Is there a type of individual who chooses academic medicine, and what values guide this person to an MD, MD/PhD, or PhD degree?

## The MD/PhD: a distinct avenue in academic medicine

The MD/PhD combined degree is a clear and relatively modern pathway in structured academic medicine. Recent reductions in Canadian MD/PhD funding cloud its future, but its historical rise has been promising. The first Canadian MD/PhD program began in 1984 at the University of Toronto, with a starting class of 2 students. It has since grown to 42 students and is the largest program of its kind in Canada ^3^. As of 2014, combined MD/PhD programs are offered at most medical schools in Canada ^4^. The number of students accepted into a given school's MD/PhD program each year varies between 1–10 across Canada, and interestingly the students are mostly men, in keeping with overall trends in academic medicine ^5^.

The demand for clinicians with different expertise, such as education, health policy, or business has attracted trainees and educators to vastly grow the training programs that are now available. Trainees may now also pursue an MSc, MBA, or similar degree at nearly any point in their career. This allows physicians to combine clinical care with an interest in managerial skills, leadership, or virtually any interest complementary to medical practice. The expansion of these academic training pathways is changing the landscape of medical research and clinical care, prompting the question of which values guide trainees to choose academic careers, and what this means for the future of academic medicine.

## Choosing academic medicine: pros, cons, and trends

There is an array of literature on the current state of academic medicine in North America ^2^. The most frequently cited disincentives are length of training, lower financial reward, and lack of autonomy ^6^. The pressure to assume the "triple threat" mantle of clinical work, research, and education can also dissuade students from academia. Program length, fear of burnout, difficulty juggling work-life balance, and advanced age at completion are also reasons for dissatisfaction ^7^. Of course, other factors such as debt and family influences also put pressure on the trainee to enter the workforce. Perhaps most concerning is that senior residents report less interest in research than junior residents over time ^7^.

Academic training that comes in addition to a 3–4 year MD degree delays the trainee’s potential professional level salaries. Until 2015, the Canadian Institutes of Health Research (CIHR) provided $21,000 (CAD) of grant funding per annum for 6 years to MD/PhD candidates ^8^. The average income 2 years after completing one’s postdoctoral degree in Canada is $65,000 ^9^. One can attempt to compare this to a second-year MD resident’s salary of roughly $60,000, with a further substantial increase upon attaining a staff position at the end of residency ^10^. Moreover, a standard MD degree takes at least 2–3 fewer years than either a PhD or an MD/PhD, therefore reducing time spent as a student.

Gender imbalance is a noted trend in academic medicine, as the overwhelming majority of those entering academics are men, and men in academic medicine have a greater salary. The salary difference between men and women is over $30,000 for early-career physician-researchers despite adjustment for work hours, specialty, and academic rank ^11^. This gender imbalance certainly needs to be addressed and rectified, both to encourage women to enter academic medicine, and to reward those that do make this decision. There are no robust studies examining the reasons for this, but a few cohort studies have shown that more women lose interest in research over time compared to men ^12^ and that women in academia were less likely to be married than those in private practice ^13^. A study with a focus group comprised of women found that perceived inflexibility in clinical pathways and decreased ability to balance competing roles were disincentives for academic medicine ^14^.

Incentives drawing a trainee towards academic medicine include a passion for research, early exposure to research, desire to become an educator, desire for clinical appointment, and strong mentorship ^2^. Most of the literature stresses that the earlier a trainee is involved in research and the more involved they are, the more likely they are to pursue academic medicine.

In terms of predicting who will enter and succeed in academic medicine, the strongest correlation is with the completion of a research fellowship or a degree such as a Masters, PhD or MD/PhD ^15^. A joint degree such as the MD/PhD is often associated with faculty and academic appointment ^15^. In a retrospective analysis of nearly 2000 medical graduates in the United States (1997–2002), those with a MD/PhD were more likely to have a full-time faculty appointment with an odds ratio of 2.33 ^16^. Publication of research conducted in medical school and residency correlated with trainees choosing academic medicine careers, as did attending a "research-intensive" university ^17, 18^.

Current trends in academic medicine include a shift of physician-scientists from laboratories to more clinical departments, and an increase in competency-based programs, which give researchers flexibility in combined programs such as the MD/PhD ^7^. In Canadian undergraduate medical education, there are numerous early academic tracks such as the MD with Special Training in Research (STiR, at the University of Alberta) and Research in Medicine (RiM, at Dalhousie University). These are aimed at cultivating an early academic interest in medical students. At the postgraduate level, there is the Clinician Investigator Program (CIP, at the Royal College of Physicians and Surgeons of Canada) for residents in sub-specialties, as well as the Clinician Scholars Program, offered by various medical schools across Canada, to both specialty and family medicine trainees.

## Values and the perspectives of individual trainees

Borges *et al.* identified three divisions of values that influence the learner’s decisions to pursue an academic career: the individual’s personal values, the values of groups with which one associates, and generational values ^6^. The only *personal value* consistently identified across studies was "intellectual stimulation" ^19^. All other personal values leading to an academic career remain poorly defined ^6^.

Meanwhile, Shea *et al.* identified characteristics of mentors and mentees that were deemed most important by a think tank of American physician-scientist programs. Personal attributes emerged at the top of both lists: for mentees the most crucial themes were passion, focus, ability to communicate clearly, desire, dedication, discipline, and resilience. For mentors the themes were prior mentoring successes, emotional intelligence, altruism, the will to promote independence, and optimism ^20^.

The following narratives from medical trainees and practicing physician-researchers attempt to shed light on the individual's personal values and the thought processes which guide career decisions towards or away from academic medicine. The five interviews were conducted by either email or telephone correspondence, from January to May 2015. The interviews were conducted, transcribed, and summarized by a single interviewer. Questions were tailored to each participant, but a basis of core questions is shown in Table 2. The participants were selected from the authors’ network of contacts to sample perspectives at each stage of academic medicine. All interviewees had completed some or all of their training at Canadian universities. The respondents consented to publication of their opinions, but were anonymized with respect to university, name, and location.

## Part 1: Considerations on prioritizing medical practice over research: the MD student perspective

### 1.1: MD involved in research

This trainee stated that he enjoyed medical research, and found it was helpful to balance this with clinical work. He also wanted to work in an academic center, and found that a research background helped to open doors in major centers. He wanted research to be a distinct but relatively minor piece in his career, and did not feel the need to do additional training. Mentorship was also key, both in getting him initially involved in research and helping him navigate the waters of academia. This MD wanted to continue his involvement with research, because he enjoyed research, the mentorship, and the career opportunities it afforded him.

According to this trainee, the biggest draw to research right now is the competitive advantage it gives for academic career positions. In major teaching hospitals, many people now need some kind of research background, and the numerous fellowships are pushing more and more people into research. Keeping them involved however, is a bit more intensive, as we need to make sure that the infrastructure and grant support is there for our clinician researchers who are already very time constrained.

### 1.2 – MSc prior to entering MD program

This trainee stated that undergraduate experiences in research inspired her to attain a Masters degree, especially in a gap between undergraduate completion and application to medical school. She later chose to pursue an MD degree as she did not envision scientific research forming the major component of her career. The student was satisfied with continued research involvement while completing her medical degree. She had confidence at this stage with her research skills as a Masters graduate. Though open to consideration of a PhD in the future, she cited length of training and concerns over a possibly diminished quality of both clinical education and research as deterrents. In particular there was a concern due to the format of an MD/PhD program, wherein gaps between the respective phases might lead to loss of clinical skills or, on the other hand, less novelty of one’s research by training’s end. This trainee personally preferred a singular focus on clinical practice as opposed to splitting her attention between that and academic medicine.

## Part 2: What an MD adds or takes away from PhD training: the PhD student perspective

This trainee stated that prior to beginning his undergraduate program, he had planned to pursue an MD. As a means to gain useful experience as an undergraduate, he became involved in a research lab. However, through his undergraduate course work, he found that he did not enjoy some activities that he thought would be critical to being successful in medical school, memorizing anatomical nomenclature and dissection labs. Concurrently, he did find that he took well to research and was able to readily follow with the logical thinking and creative problem solving that are fundamental to academic research. This PhD student has since continued his research career as a postdoctoral research fellow, and plans to work towards a professor position at a research-intensive university. He commented that not having an MD and its related training have posed some limitations to his work, as he can not readily recruit patient populations without first finding an MD with relevant background and interests to collaborate with.

While he does not think that everyone interested in obtaining an MD would be better suited with a MD/PhD, he does think that scientific literacy is an important skill for MDs, and that becoming involved in a research laboratory at the undergraduate level should be encouraged for students considering a medical career, even if just in volunteer capacity. Exposure to academia may make future doctors more readily able to incorporate scientific advances into their practice.

## Part 3: Balancing practice and research: the MD/PhD student perspective

This student chose the MD/PhD path because of the inspiring early undergraduate research experience she was involved with. These experiences ranged from volunteering in a laboratory doing benchwork, to interacting with patients in clinic. When she had a gap between undergraduate completion and application to medical school, her strong MD/PhD mentors, including the Program Director, guided her towards academic medicine.

In terms of personal values, this trainee had a strong sense of social justice and felt that her research was emotionally as well as intellectually stimulating. She felt that her attempts to effect large-scale change would be eased by having the MD/PhD degree. This was a way for her opinions to carry more weight, which would strengthen her ability to advocate for her patient population. As someone who was planning to spend half her future career in research and half in clinical duties, she was overall very well suited to her program.

Regarding the gender divide, she noted that although numbers were equal for men and women early in the program she found fewer mentors who were women on the tenure track and at academic conferences. Another personal value in her decision was her relatively young age at entry to the program. She felt that the MD/PhD path gave her more time to decide what field of medicine to devote herself to, and this was time she could afford.

## Part 4: Practicing MD returns to pursue a PhD: the lifelong-learner perspective

This experienced physician felt that his practice situation, at a community health centre, had stabilized over the years thanks to a team of seasoned colleagues. Now was a good time to ease up on clinical commitments and pursue a PhD in philosophy. From a personal perspective, he reflected on returning to academia having something to do with his time of life. He wished to continue intellectual challenges, broadening his understanding and knowledge, as well as analytic and argument skills. As he serves a low socioeconomic status population, he felt he could improve his value as a resource with this formal training in philosophy – by advocating more effectively. He cited his belief that we need to ask ourselves honestly what we owe one another in this world, a broad question that could not be answered within the narrow confines of medical practice. He envisioned teaching, mentoring and modeling a more pervasive and broadly-informed understanding of philosophy, morals and values as applied to the healthcare and other systems. He wished to place particular emphasis on a more complex and complete understanding of marginalized populations, and of the role of the physician in society. In seeing how society treats its marginalized sections, as well as many years of coming up against healthcare’s attitudes toward disadvantaged populations, he was convinced of the necessity to ask questions in a different way. A PhD in philosophy would help this physician formulate those questions to be more clear, meaningful, and effective.

## Discussion

While these are only a handful of individuals’ perspectives, in aggregate they provide insight into the considerations involved in pursing academic medicine, as well as the benefits of formal research training to medical practice. Each of these trainees pursued further academic training at different times in their careers, drawn to academia by a passion for research, critical thinking, social justice and the influence this would have on their career path ( Table 1). Similarly, many trainees also felt that time duration and financial constraints were drawbacks of pursuing research. This is extremely relevant now, given the recent funding changes in the Canadian Institute of Health Research for MD/PhD programs. Namely, as of 2016, the CIHR has discontinued funding of their thirty-year old MD/PhD studentship program, in universities across Canada ^21^. Though an MD/PhD is not for everyone, it is unfortunate that this program has been cancelled, likely resulting in a decrease in crossover between medical practice and research. Undoubtedly, the MD/PhD programs in Canada cannot continue as they were before.

**Table 1.** Perceived draws and deterrents related to a career in academic medicine.

Draws of academic medicine	Deterrents from academic medicine
**Passion for research**	Prolonged training
**Desire to educate**	Lower income
**Impact of a strong mentor**	Reduced autonomy
**Desire for academic** **appointment**	Family considerations

**Table 2.** Core questions posed in email and telephone interviews with participants.

1. Why did you choose to pursue an MD, MD/PhD or PhD at this stage?
2. What were some personal values going into this decision?
3. What further academic (either research or education) career do you envision for yourself? Will your ongoing/future practice be purely clinical, purely research-centred, or a blend of both?
4. Can you comment on the impact of mentors (either positive or negative) towards your decision to get an MD vs an MD/PhD vs PhD?
5. Are you satisfied with your current degree of academic involvement?
6. Do you feel that your university/work environment is a highly-supportive culture for academia? Do you consider it "renowned" for academics, and did that influence your decision to get your MD vs MD/PhD vs PhD?
7. Were financial reasons part of why you decided to be an MD vs PhD vs MD/PhD? What about length of program/time constraints?
8. (If applicable): do you have any thoughts on being female and the career decisions you have made towards or away from academia?

Funding matters aside, there is an interesting gender difference in terms of academic medicine engagement. Multiple studies and focus groups have tried to characterize the values and reasons behind the relative lack of trainees and mentors who are women in academic medicine ^11– 14^. This warrants further attention as women likely share the same personal values as their colleagues who are men, that will draw them to academic medicine, yet additional deterrents have been identified within this group.

## Conclusion

The personal values that draw one to academic medicine can be used to improve recruitment to academic programs. More research is needed on definition and classification of these personal values, but “intellectual stimulation” is the most consistently identified. Trainees that have a passion for research and academic advancement can be encouraged along this path by identifying them and pairing them with a strong mentor early in their careers. All trainees emphasized the value of mentorship in academia, and its foundation in making or breaking a career in research. Alternatively, we should also be aware of the very real deterrents that are turning away qualified trainees. Long training periods, a lack of autonomy, and financial considerations were identified as deterrents from a career in academic medicine. Being aware of these perceived barriers allows policy makers to address them and help to recruit the best trainees through modification of existing programs.

At a time when the public’s knowledge of basic science and fundamental medicine is lagging relative to the rapid development of medical science, technology, and social policy ^22^, those who pursue academic medicine will be the essential communicators who bridge the gap. It is paramount that these individuals and groups are identified, supported and lauded for their intellectual thirst. Understanding the personal values and constellation of factors which help an individual decide on a career in academic medicine will hopefully streamline access to academic positions which suit the trainee. This in turn will likely produce medical, scientific, and health policy advancements which will efficiently shorten the “knowledge translation” gap. Overall, this bodes well for patient care at the individual level and for society at large.
