# Supplementary material for: Personal values influencing career path in academic medicine: Perspectives of selected Canadian trainees
Source: F1000Res. 2018 Apr 25;5:1903. Originally published 2016 Aug 2. [Version 2] doi: 10.12688/f1000research.9026.2 (PMC6092908; doi:10.12688/f1000research.9026.2)
Supplement: Peer review history

# Peer review: Referee response for version 2

**DOI:** 10.5256/f1000research.16068.r36944

**Published:** 2018-08-10

**Authors:** Jens Foell

**Affiliations:** 1 Department of Psychology, Florida State University, Tallahassee, FL, USA

The presented article is a summary of current findings regarding the driving factors in deciding to pursue MD training, PhD training, or both. Based on findings in the cited literature, the article discusses a psychological drive for intellectual stimulation, together with available mentorship, as the most consistent drivers of these decisions. Examples for different career paths are presented, based on interviews that the authors have conducted. All of this is presented from a Canadian / North American perspective.

 The article is very well written and the topic is timely and should be interesting and useful to a large number of readers. While the article is very good as it is, I would like to raise a few discussion points in the hope that they might improve the manuscript further:

1. As mentioned above, the presented information is given in the context of the Canadian / North American system and the authors make this clear at several points in the text (as well as in the title) so as not to create any confusion. However, for a reader outside of this region, it might be useful to know that much of the information given in the text might be different for them: not all countries offer combined MD/PhD career paths, and/or the interplay between clinical work and research might be structured differently. Similarly, funding structures differ between countries, and the decision to go in a specific direction might have to be made earlier or in a different context than in the given examples. I don't think that all of this needs to be described here, as it would distract from the main points of the text, but I think it would be useful to mention, either in a footnote or in the Discussion section, that the influence of personal values as described here might conceivably be different in an international context, where other/additional influential factors might be at play.
2. I very much appreciate the authors addressing the gender imbalance in academia, which is certainly an influencing factor on individual career decisions. This is similar to the situation for people with a low socioeconomic background or those belonging to an underrepresented minority (URM). Now, it is not my intent to dilute the very important discussion of gender issues by introducing another topic that will then compete for the reader's attention. However, it does feel that its omission makes the article miss an important additional influential factor of career paths and decisions, especially since discouragement is being discussed. My recommendation would be to mention this topic in a footnote or in the discussion section, making the point that, similar to the mentioned gender imbalance, belonging to an URM or coming from a non-academic or low-socioeconomic background can present a filter, limiting one's actual or perceived options regarding academic career paths. These filters might not act in the same way, to the same degree, or at the same entry points as the mentioned gender imbalance, and they might not have been studied to the same degree. Nevertheless, they might interact with the mentioned deterrents to limit options and to otherwise impede the decision-making process.
3. A minor note: it is mentioned that women in academia are less likely to be married than those in private practice (citation 13). Were they also less likely to be married than men in comparable positions? I assume this is the case; either way it might be an interesting addition to this sentence, maybe in parentheses or as a footnote.

Thank you very much for writing this important and interesting article.

I have read this submission. I believe that I have an appropriate level of expertise to confirm that it is of an acceptable scientific standard.

# Peer review: Referee response for version 2

**DOI:** 10.5256/f1000research.16068.r33426

**Published:** 2018-05-09

**Authors:** Tobias C. Wood

**Affiliations:** 1 Department of Neuroimaging, Institute of Psychiatry, Psychology & Neuroscience (IoPPN), King's College London (KCL), London, UK

I have no further comments.

I have read this submission. I believe that I have an appropriate level of expertise to confirm that it is of an acceptable scientific standard.

# Peer review: Referee response for version 1

**DOI:** 10.5256/f1000research.9710.r20199

**Published:** 2017-02-27

**Authors:** Tobias C. Wood

**Affiliations:** 1 Department of Neuroimaging, Institute of Psychiatry, Psychology & Neuroscience (IoPPN), King's College London (KCL), London, UK

This opinion article consists of two parts.

 The first is a thorough literature review. It gives a solid overview of the issues surrounding the MD/PhD program. I have no issues with this section.

 The second part consists of five transcribed interviews, and I consider it weaker than the first section. The evidence presented is only anecdotal, with a very small sample size. The author states that the interviewees were drawn from the author's academic contacts, so although a range of views and backgrounds is represented, sample bias cannot be excluded. Given that this an opinion and not a full research article this is not a reason for rejection, but this section could be greatly strengthened with additional interviews, ideally from outside the author's network. Alternatively, given the length of time that has elapsed since the original interviews, follow-up interviews checking whether the participants still felt the same way about their courses would be enlightening.

 The discussion and conclusion sections are mostly good. However, the second paragraph of the discussion contains the following sentence: "Multiple studies and focus groups have tried to characterize the values and reasons behind the relative lack of female trainees and mentors in academic medicine.", but no citations are given. I suspect that the authors are correct in this assertion, but a relevant citation or other evidence to back up this statement must be added before indexing.

I have read this submission. I believe that I have an appropriate level of expertise to confirm that it is of an acceptable scientific standard, however I have significant reservations, as outlined above.

# Peer review: Referee response for version 1

**DOI:** 10.5256/f1000research.9710.r20201

**Published:** 2017-02-24

**Authors:** Kirstie J. Whitaker

**Affiliations:** 1 Alan Turing Institute, London, UK

**Synopsis**

 Tsoi and colleagues start with a definition of academic medicine and a summary of this career path and who chooses to take it. They then explain the pros and cons of academic medicine as have been described in the currently published literature. Finally, they present summaries of interviews conducted with five medical professionals: two MD students, one PhD student, one MD/PhD student and one experienced clinician (MD) who has returned to complete a PhD at an advanced stage of his career.

**Compliments**

 It is important for personal motivations and values to be included in our understanding of career paths in all aspects of science and medicine. This article has the potential to take “common knowledge” and hearsay and put it into a peer-reviewed literature. It makes these discussions findable and citable: important steps towards progressing our understanding of addressing structural inequalities in career progression. This work stands to benefit many readers who are interested in careers in academic medical but are looking for more detailed information on the prospect.

**Concerns/Suggestions**

*Audience*

 Who is the intended audience for this opinion piece? The abstract mentions trainees, educators and policy makers, and the authors are right that this topic is of interest to all these groups. Unfortunately, the focus for each of them are quite different and I think the paper fails to provide succinct take home messages for any of them. (Alternatively, this is a qualitative research article and should be structured as such, see my comments below).

 I’d recommend developing the discussion section and potentially including actions that interested parties may take as a result of the findings presented in the paper. To be clear: my recommendation is to restructure the opinion article, as there is a lot of useful advice/information included in the introduction that I think could be put to better use supporting the research findings, rather than add in more information to the discussion.

*Integrating personal values*

 The introduction to this opinion article outlines most of the arguments for/against a career in academic medicine so the summaries of the interviews do not end up providing much new information to the reader. If the point of the article is to present personal values, I’d recommend integrating quotes from the interview subjects along with the background literature. Although not necessary, I suspect that arranging the information around themes rather than by individual participant would synthesise the findings more effectively. Qualitative research is not my area of expertise but I enjoyed these suggestions from London School of Economics (which includes links to further reading on the topic).

*Missing voices*

 There are key demographics who are not interviewed in this article on the career path in academic medicine: specifically, those of clinicians who do not see the benefits of it and/or who those who were not able to continue along this path. Furthermore, all people interviewed are students which leaves out most of the time spent along this career path. This is fine, but I’d like the authors to acknowledge that they are presenting a biased sample which may limit its generalisability for readers considering a career in academic medicine.

 Related to this point, and more minor in my opinion, is that this article seems to be written about academic medicine careers in Canada. I’d recommend that the authors clarify this focus in the abstract and either acknowledge that these findings may not be relevant to clinicians in other countries or develop the breadth of the introduction and discussion to consider these differences.

*Females*

 Please do not refer to adult women as “females”. The use of this adjective as a noun reduces women to their reproductive abilities and diminishes their humanity (see Jezebel for my favourite article on the subject). I’m confident that this was not the authors’ intention, but given that a focus of this opinion article is the reasons women are not well represented within academic medicine, I’d advise avoiding further alienation. I’d recommend referring to women and men (rather than males) as appropriate.

I have read this submission. I believe that I have an appropriate level of expertise to confirm that it is of an acceptable scientific standard, however I have significant reservations, as outlined above.
